# Preocular sensor system for concurrent monitoring of glucose levels and dry eye syndrome using tear fluids

**DOI:** 10.1371/journal.pone.0239317

**Published:** 2020-10-07

**Authors:** Jae Hoon Han, Yong Chan Cho, Won-Gun Koh, Young Bin Choy

**Affiliations:** 1 Interdisciplinary Program in Bioengineering, College of Engineering, Seoul National University, Seoul, Korea; 2 Department of Chemical and Biomolecular Engineering, Yonsei University, Seoul, Korea; 3 Institute of Medical & Biological Engineering, Medical Research Center, Seoul National University, Seoul, Korea; 4 Department of Biomedical Engineering, Seoul National University College of Medicine, Seoul, Korea; University of Salamanca, SPAIN

## Abstract

The present study demonstrated a noninvasive preocular sensor system for the concurrent monitoring of diabetes and one of its prevalent complications, dry eye syndrome (DES), using tear fluids. Two distinct sensors, i.e., the glucose and DES sensors, were prepared and encased together in a single housing unit to produce the sensor system, and the tip was designed to be in contact with the eye surface noninvasively to collect and deliver tear fluid to the sensors. The glucose sensor was modified from a commercially available electrochemical sensor to allow for the measurement of glucose concentrations, even in a small amount of collected tear fluid. The DES sensor was equipped with a microchannel spaced with two parallel electrodes to determine the amount of collected tear fluid. *In vivo* experimental results revealed that with the collected tear fluid of about 0.6–1.0 μl, the sensor system estimated the blood glucose concentrations with acceptable accuracy compared with that of the glucometer in clinical use. The DES condition in animals was diagnosed with high sensitivity (91.7%) and specificity (83.3%).

## Introduction

Diabetes is one of the most common chronic diseases, with an estimated 422 million people affected worldwide in 2014 [1, 2]. One of the major complications in diabetic patients include dry eye syndrome (DES), which is a disease characterized by dysfunction in tear production or tear film instability [3–6]. More than 50% of diabetic patients are also diagnosed with DES, which shows a significant correlation with the duration of diabetes. Wounds tend to heal more slowly with diabetes, and thus, it is important to prevent any preocular damages caused by DES [3]. Therefore, more frequent monitoring of blood glucose levels and DES would be beneficial for diabetic patients.

Diabetic patients are recommended to measure their blood glucose concentrations multiple times daily, especially after meals [7, 8]. Blood is withdrawn somewhat invasively by pricking the fingertip with a needle, which may cause psychological trauma and form a callus at the needled site, which lowers patient compliances [9]. DES is diagnosed clinically using the Schirmer’s test, which measures the volume of basal tear fluid by collecting it in a paper strip that is in contact with a sensitive preocular surface for a relatively long time of minutes [10, 11]. To be accurate, the generation of reflex tear fluid needs to be minimized, and thus, the Schirmer’s test is often performed by a healthcare professional [12, 13].

Therefore, a system that allows for minimally invasive ease-of-use measurement of glucose levels and the amount of basal tear fluid would be advantageous given the need for the chronic management of diabetes and its highly relevant DES. Therefore, measurements using tear fluid may be an advantageous strategy. There is a potential correlation of glucose levels between tear fluid and blood serum in diabetics [14–16]. The collected volume of basal tear fluid itself is already a diagnostic measure for DES, as performed in the Schirmer’s test [17]. Therefore, basal tear fluid may be carefully collected to concurrently measure its amount and glucose concentration to monitor DES and diabetes, respectively, instead of creating puncturing wounds for blood extraction. However, previous methods often required the collection of a relatively large volume of tear fluid [18], and the tear collectors that must be in contact with the eye surface primarily contained sharp edges and corners [19, 20]. Therefore, these collectors likely induce reflex tear fluid and ocular tissue damage during the collection process.

To resolve these problems, we propose a sensor system equipped with a minimally invasive tear collector to simultaneously monitor diabetes and DES. For glucose measurement, a commercially available electrochemical sensor of strip shape (Accu-Chek Performa test strip, Roche Diagnostics, Rotkreuz, Switzerland) was used and modified to allow for measurement even in a small amount of collected tear fluid [18]. For the diagnosis of DES, we fabricated a separate strip-type sensor equipped with a micron-scale channel for tear collection, and the amount of collected tear was detected using a pair of electrodes that measured the change in electrical resistance caused by tear infiltration into the channel. These two different sensors were assembled and embedded in a single housing unit of the system, which was designed to be minimally invasive for tear collection. Therefore, the tip to be in contact with the eye surface was shaped to have a proper area and round edges to avoid any possible damage to the eye surface [21]. The system was purposed to be in contact with a less sensitive eye surface, the inferior palpebral conjunctiva (IPC).

The performance of the system embedded with two different sensors was first evaluated under *in vitro* environments using artificial tear fluid with varied glucose concentrations. The artificial tear fluid was flowed on a glass surface at different flow rates to form liquid films of different thicknesses to simulate tear films under normal and DES conditions. For *in vivo* evaluations, the sensor system was tested using rabbit eyes. To induce a DES model, the animal eyes were pretreated with a topical administration of atropine sulfate eye drops [17]. To induce diabetic conditions, the blood glucose level was increased via anesthesia using ketamine and xylazine [19, 22].

## Materials and methods

### Sensor system design and fabrication

**Fig 1:**
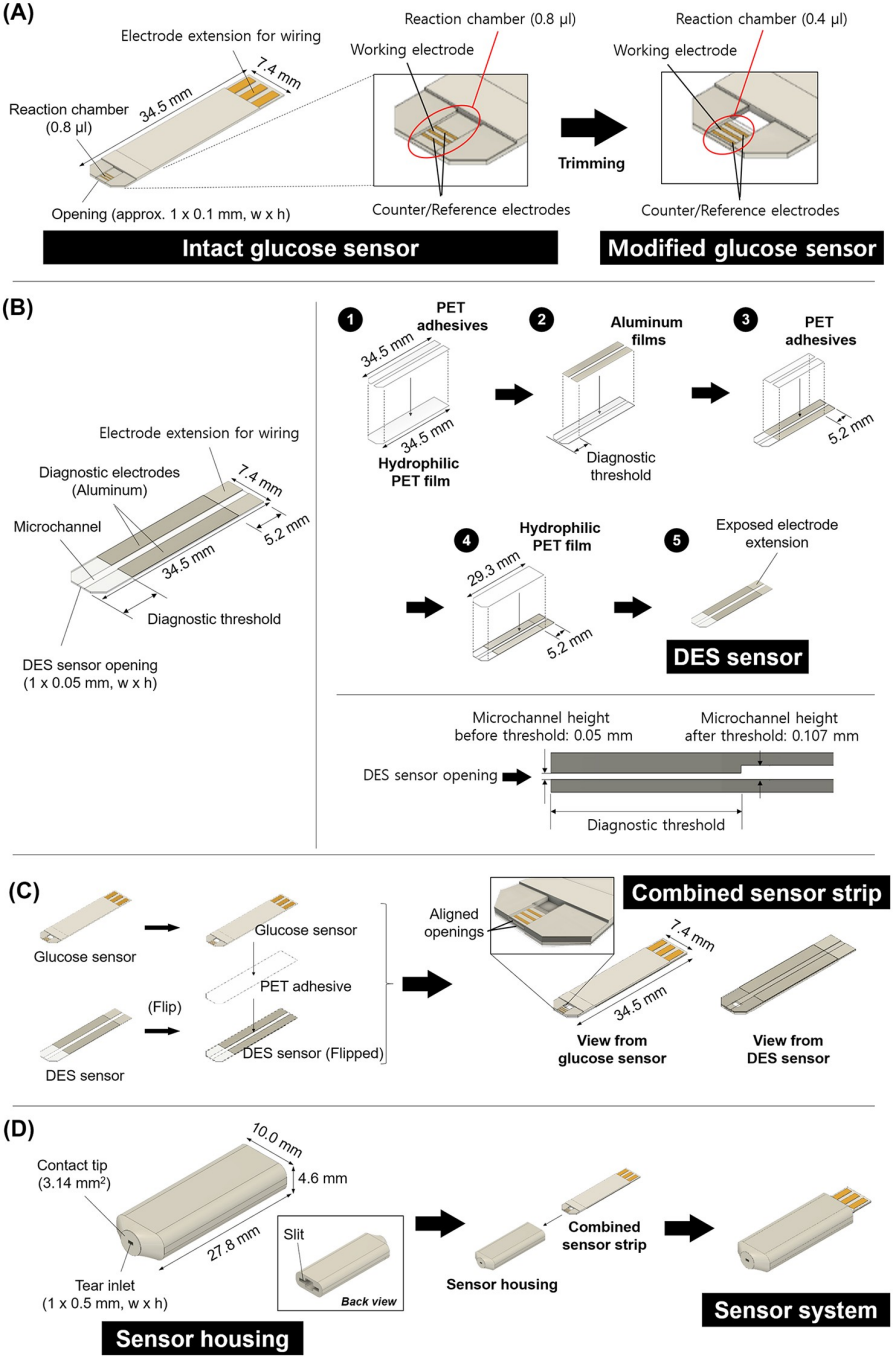
Schematic fabrication procedures of (A) glucose sensor, (B) DES sensor, (C) combined sensor strip, and (D) final sensor system. The images were drawn by the authors using Solidworks (SOLIDWORKS Standard 2017 Research, Dassault Système, Vélizy-Villacoublay, France).

We first prepared two separate strip-type sensors for concurrent measurement of glucose concentrations and volumes of tear fluids, i.e., for diagnosis of diabetes and DES, respectively. For a glucose sensor, a commercially available strip-type of electrochemical glucose sensor, an Accu-Chek Performa test strip, was modified as depicted in our previous work (Fig 1A) [23]. Briefly, the Accu-Chek Performa test strip is originally equipped with a reaction chamber to collect 0.8 μl of blood, which was cut and reduced to 0.4 μl to allow for the collection of a small volume of tear fluid. During this modification, we retained the necessary measuring electrodes, such as the counter, reference and working electrodes.

A DES sensor was designed to collect tear fluid through a microchannel, where we determined the DES by setting a diagnostic threshold of the volume of the collected tear fluid. A microchannel was made of hydrophilic PET films to facilitate the absorption of tear fluid via capillary pressure in a determined, short time of approximately 10 s (Fig 1B). To assess whether the volume of collected tear fluid exceeded the diagnostic threshold, a pair of the diagnostic electrodes were embedded to be in contact with the microchannel. The length of the diagnostic electrodes was determined based on the *in vivo* experimental results obtained using the DES sensor without the electrodes (i.e., the DES sensor (W/O)) [24].

The DES sensor was fabricated to have the same dimension (34.5 mm x 7.4 mm x 0.4 mm, l x w x h) as the glucose sensor in this work. A hydrophilic PET film, 0.1 mm in thickness, was cut into the dimension of 34.5 mm x 7.4 mm (l x w) as a bottom layer, and a pair of PET adhesive tapes, 0.05 mm in thickness, were attached on top with a 1-mm gap between each other to form a microchannel. A pair of aluminum films, 0.007-mm in thickness, were attached on the PET adhesives at a length from the diagnostic threshold to the end of the adhesive tapes to serve as diagnostic electrodes. These electrodes were omitted in preparation of the DES sensor (W/O). A pair of PET adhesive tapes were applied on top of those films at a length that was 5.2 mm shorter than the aluminum film to expose the conductive aluminum films and make them an electrode extension for wiring. Finally, a hydrophilic PET film, again 5.2 mm shorter than the bottom layer, was attached as a top layer to cover the sensor and expose the electrode extension. The cross-section of the microchannel in this assembly was 1 mm x 0.05 mm (w x h) from the opening to the diagnostic threshold, and it increased to 1 mm × 0.107 mm (w x h) to the back of the sensor.

We assembled the glucose and DES sensors together by bonding the bottom layers with an adhesive tape to produce a single combined sensor strip (Fig 1C). We aligned the openings of both sensors to allow for the concurrent collection of tear fluid. The assembled sensors were inserted into a sensor housing to produce a sensor system. The sensor housing was designed and fabricated using SolidWorks software and 3D printer, respectively (Fig 1D). At the front of the housing, we prepared a slightly protruded contact tip that had a round shape without sharp edges and an area of 3.14 mm^2^ to allow noninvasive contact with the IPC [21]. At the contact tip, an inlet for tear collection was formed with a cross-section of 1 mm × 0.5 mm (w × h). At the back of the housing, a slit was made to tightly insert the combined sensor strip in a way that the sensor openings could reach and align with the inlet, and the electrode extension could be exposed for wiring.

### *In vitro* experiments

*In vitro* evaluation of the sensor system was performed using a simulated biological fluid that mimicked the non-Newtonian property and viscosity of tear fluid [25]. Therefore, we first prepared an artificial tear fluid with the addition of 2 mg/ml lysozyme and 1.65 mg/ml lactoferrin to phosphate-buffered saline (PBS) at pH 7.4. D-(+)-glucose at varying concentrations of 0, 0.9, 1.8, 9 and 18 mg/dl was added to the resulting artificial tear fluid to simulate the possible range of tear glucose concentrations that corresponded to plasma glucose concentrations [26, 27]. To simulate normal and DES conditions, we prepared the films of the artificial tear fluid with two distinct thicknesses (S1 Fig). The artificial tear fluid was supplied on a glass surface that was 1 mm in width and 4 cm in length at a flow rate of 8 μl/min and 5 μl/min to create the tear films that were 14.8 ± 2.1 μm and 8.6 ± 1.4 μm in thicknesses to simulate the known tear thicknesses under normal and DES conditions, respectively [28]. For all of the glucose concentrations used in this work, the artificial tear fluid exhibited a similar thickness profile for each different flow condition.

For *in vitro* evaluation, the sensor system equipped with the DES sensor (W/O) was tested under 10 different conditions (i.e., 5 different glucose concentrations and 2 different film thicknesses). For each condition, 4 different sensor systems were tested, where the tip of the sensor system was in contact with the tear film for 10 s. The extensions of the glucose sensor electrodes were connected to a potentiostat at 150 mV to measure the electric currents, and an equation was generated for calibration to the corresponding known glucose concentrations [29, 30]. We also measured the length of tear fluid that infiltrated into the microchannel in the DES sensor (W/O) using a Vernier caliper (Mitutoyo, Kanagawa, Japan).

### *In vivo* experiments

The *in vivo* experiments were approved by the Institutional Animal Care and Use Committee (IACUC No. 18–0253) at the Biomedical Research Institute of the Seoul National University Hospital. All experiments were performed under relevant guidelines and regulations. Male New Zealand white rabbits (2.5~3.0 kg) were raised in a controlled environment (temperature: 21 ± 1°C, humidity: 55 ± 1%, light/dark cycle: 12 hours, and food and water ad libitum). The animals were assigned to two different groups, i.e., the animals with normal eyes and DES, respectively, and only the right eye was used for each animal. To induce DES, a 50 μl drop of a 1.0% sulfate solution was applied to the lower cul-de-sac of the right eye at 8.00 a.m., 1.00 p.m. and 6.00 p.m. every day for 4 days (S2 Fig) [17]. The treated eye was evaluated using the Schirmer’s test, where tear absorption length less than 8 mm was considered as the animals with DES [17, 31].

We first used the sensor system equipped with the DES sensor (W/O) and measured the length of tear fluid infiltration into the microchannel, as performed under the *in vitro* testing environments. For this test, 17 and 16 animals under normal and DES conditions were used, respectively. Therefore, the tip of the sensor system contacted the IPC for 10 s, and the data of infiltration length were assessed to set a diagnostic threshold (i.e., the location and length of the diagnostic electrode in the DES sensor) based on the sensitivity and specificity [24].

We then fabricated and tested a final sensor system, which was embedded with the glucose sensor and the DES sensor equipped with the diagnostic electrodes. For the normal and DES animal groups, we also increased the blood glucose level via a subcutaneous injection of a cocktail of 15 mg/kg ketamine and 5 mg/kg xylazine, as reported in previous studies (S2 Fig) [23, 26]. At scheduled times of 0, 30 and 60 min after injection of anesthetics, the sensor system was applied to the rabbit eye, as described above. Four eyes were tested at each scheduled time of tear collection for each animal group. The extension of the glucose sensor electrodes was connected to a potentiostat at 150 mV to measure the electrical current, which was calibrated with the equation obtained from the *in vitro* tests to give the actual glucose concentration in tears. The actual glucose concentration in blood was also measured at the same time of tear collection. Blood was withdrawn from the right ear vein of the rabbit and measured using a glucometer. For DES diagnosis, the extension of the DES sensor was connected to a digital multimeter to measure the electrical resistance.

## Results

### Sensor system fabrication

**Fig 2:**
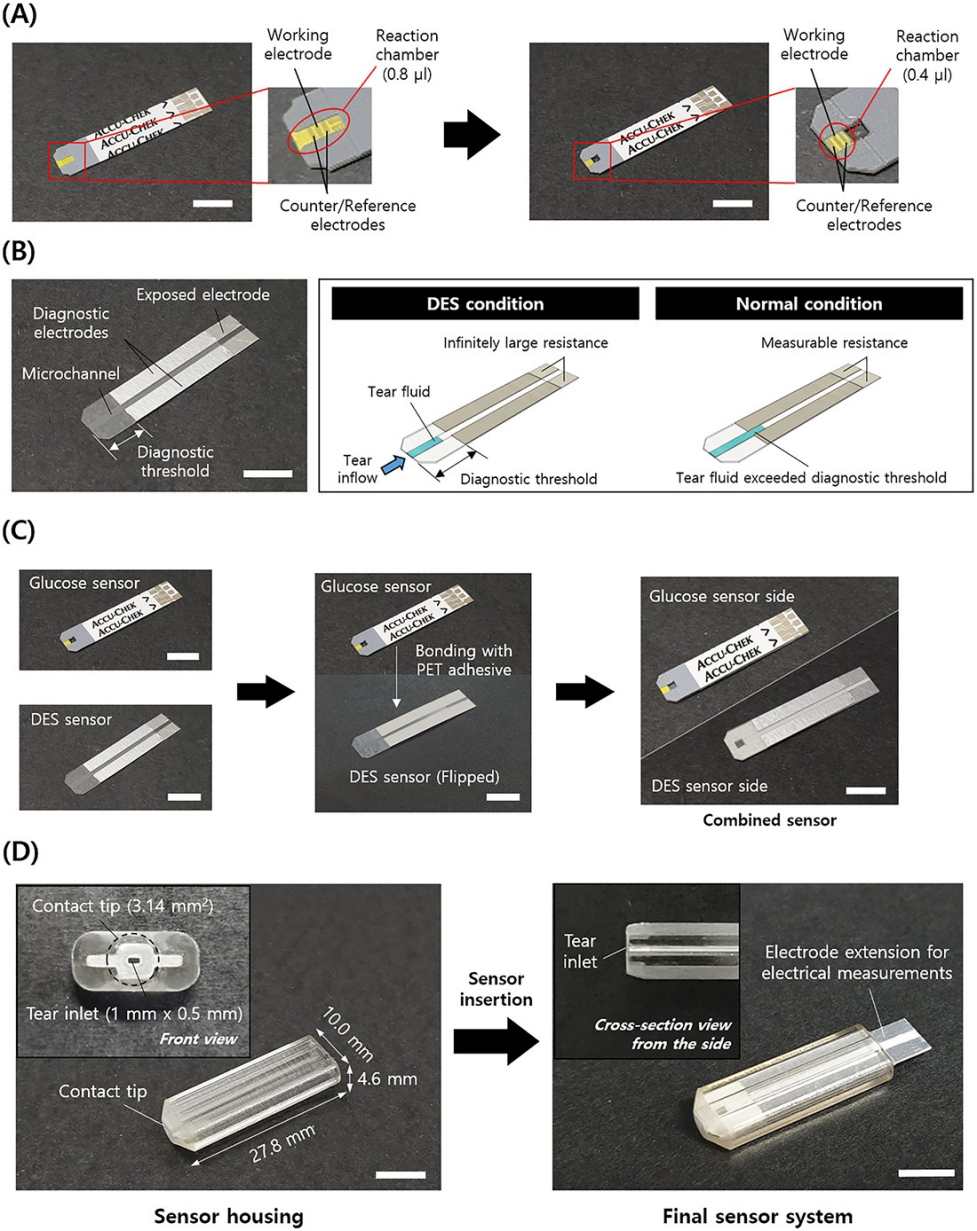
Optical images of (A) glucose sensor, (B) DES sensor, (C) combined sensor strip, and (D) final sensor system. The images were drawn by the authors using Solidworks (SOLIDWORKS Standard 2017 Research, Dassault Système, Vélizy-Villacoublay, France).

Fig 2 shows the optical images of the sensor system and its constituent units, which were fabricated as depicted in Fig 1. The volume of the reaction chamber in the glucose sensor was properly reduced, and the necessary electrodes remained unaltered. The DES sensor was also successfully prepared to possess a microchannel and diagnostic electrodes. Therefore, the electrical resistance was measurable or infinite when the tear fluid infiltrated up to the point after and before the diagnostic threshold, respectively (Fig 1B). These two sensors were attached as a single strip by bonding their bottom sides with alignment of their openings, which was tightly inserted into the slit in the sensor housing to produce the sensor system herein.

**Fig 3:**
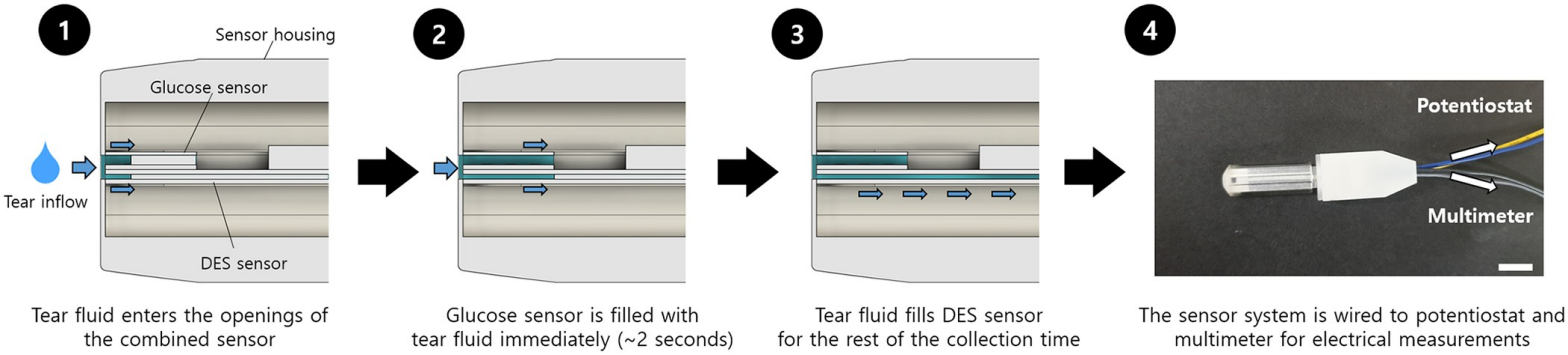
Description of tear collection scenario and measurements. The images were drawn by the authors using Solidworks (SOLIDWORKS Standard 2017 Research, Dassault Système, Vélizy-Villacoublay, France).

Fig 3 depicts the tear collection process with the sensor system. In this work, the contact tip of the sensor system was in contact with the IPC of the eye for 10 s. Therefore, right after contact, tear fluid was absorbed through the inlet and delivered concurrently to the openings of the two sensors, i.e., the glucose and DES sensors prepared in this work. During this process, the reaction chamber in the glucose sensor was filled more rapidly (< 2 s), and the fluid was continuously infiltrated into the microchannel of the DES sensor for the entire contact time of 10 s. The sensor system was freed from the eye surface, and it was wired to a potentiostat and multimeter for measurements of electrical current and resistance, respectively, which were calibrated to determine the glucose level and DES condition, respectively.

### *In vitro* evaluation

**Fig 4:**
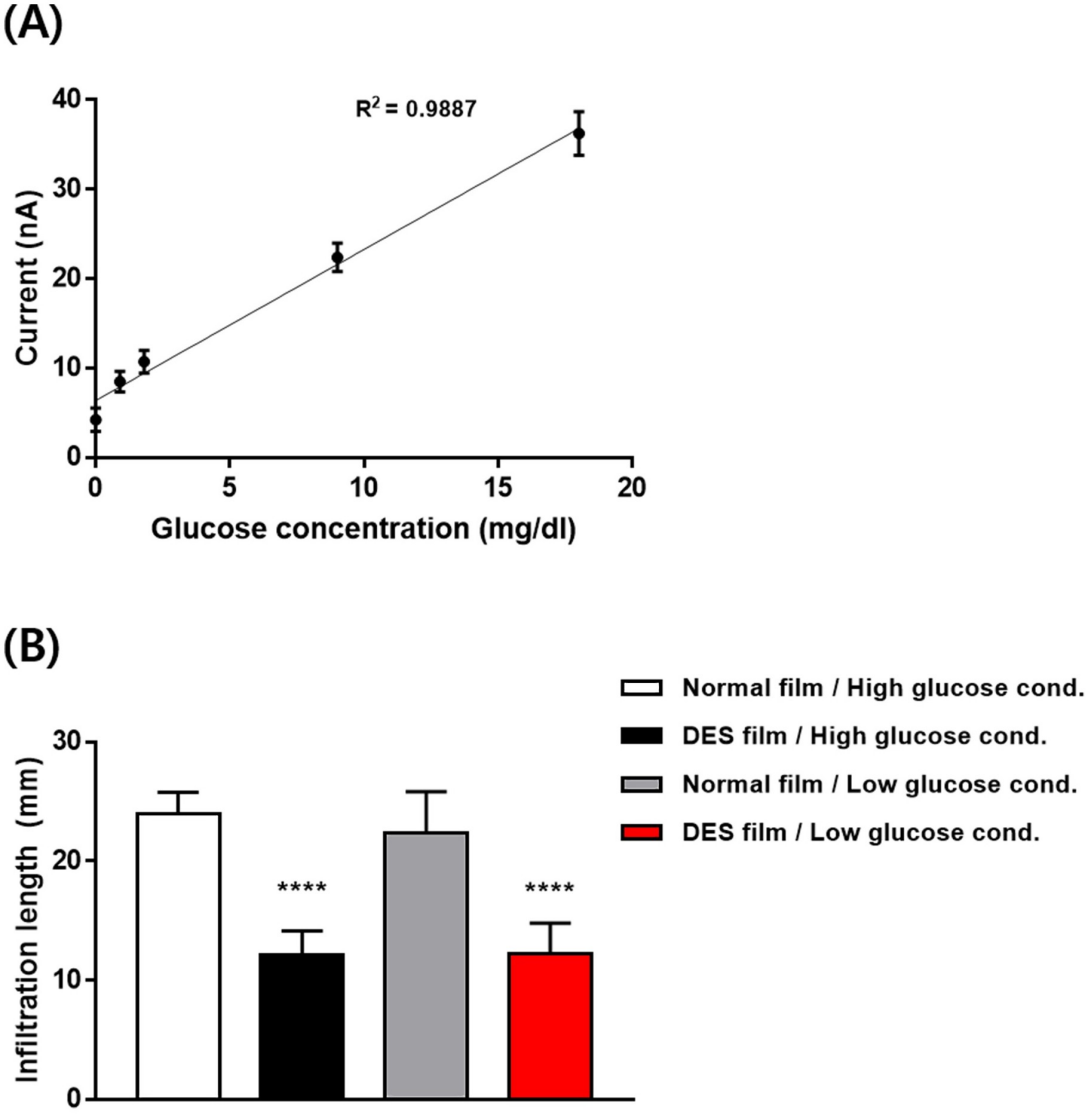
*In vitro* evaluation of the sensor system. (A) Plots between the measured electrical current and known glucose concentrations. (B) Infiltration length of collected tear fluid measured under *in vitro* simulated conditions. **** statistically significantly different from all normal films (Student’s t-test; *P* < 0.0001).

For *in vitro* evaluation, we first used the sensor system embedded with the glucose sensor and DES sensor (W/O). A tear film of two different thicknesses was prepared using an artificial tear fluid to simulate the normal and DES conditions, respectively (S1 Fig). For each thickness, the glucose concentrations were varied to calibrate the glucose sensors in the system. When the sensor system was in contact with the simulated tear film, both sensors absorbed sufficient tear fluid for measurements. As shown in Fig 4A, the glucose sensor showed a highly linear relationship between the values of the electrical current and glucose concentration (Fig 4A), irrespective of the tear film thicknesses (S3 Fig). Given these results, we generated the following equation to calibrate the glucose concentration in tears for *in vivo* evaluation:

Glucoseconcentration(mg/dl)=0.59(mg/dl/nA)×Current(nA)−3.76(mg/dl)

For the DES sensor (W/O), we assessed the infiltration length for four different conditions: 1) normal film and low glucose conditions (i.e., 0, 0.9 and 1.8 mg/dl), 2) normal film and high glucose conditions (9 and 18 mg/dl), 3) DES film and low glucose conditions, and 4) DES film and high glucose conditions. As shown in Fig 4B, the infiltration length from the DES film was statistically significantly lower than normal film. However, for each film, the difference was not observable between the two different glucose conditions, the range of which represent the tear fluids with low/normal and high blood glucose levels (Fig 4B) [27]. This result suggests the presence of a threshold between the volumes of collected tear fluid under the normal and DES conditions.

### *In vivo* evaluation

**Fig 5:**
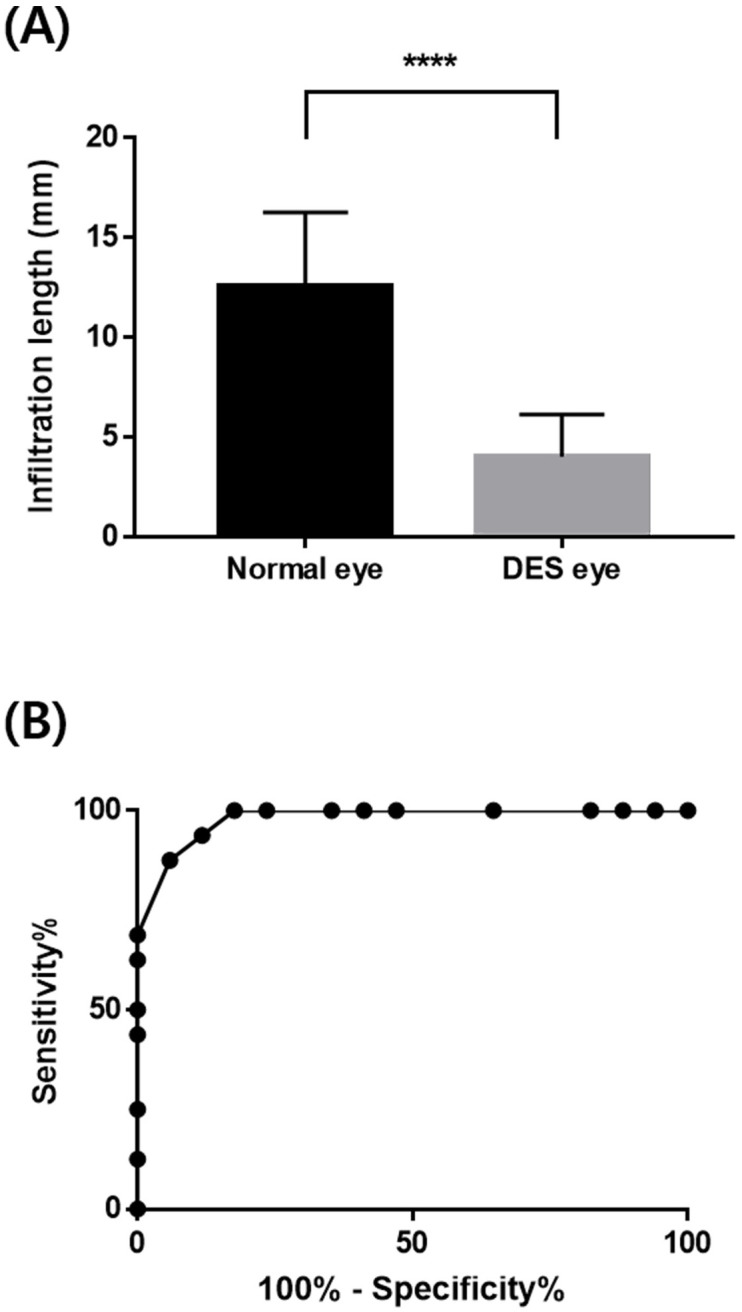
Infiltration length assessed for determination of the diagnostic threshold. (A) Profiles of tear infiltration length and (B) ROC curve obtained from normal and DES eye conditions *in vivo*. ****P < 0.0001.

We first sought to identify the diagnostic threshold using the sensor system with the DES sensor (W/O), where the infiltration length of tear fluid was assessed using rabbit eyes of normal and DES conditions. As shown in Fig 5A, there was a significant difference in infiltration length of the collected tear fluid between the normal and DES eyes, which were measured as 12.56 ± 3.72 mm and 4.03 ± 2.12 mm, respectively. Therefore, considering the tear volume of 0.4 μl collected in the glucose sensor, the total volumes of collected tear fluid for a whole sensor system would be about 1.0 and 0.6 μl for the normal and DES eyes, respectively. Given with those data, we plotted a receiver operating characteristic (ROC) curve because the DES sensor was purposed to work as a binary classifier to determine the presence of DES condition [32]. As shown in Fig 5B, the area under the curve was close to 1 (0.9798), which suggested that the strategy with the DES sensor possessed an acceptable diagnostic capacity to distinguish the normal and DES eyes. To determine the diagnostic threshold, we assessed the diagnostic odds ratio (DOR) using the following equation [24]:

$$\text{DOR}=\;\frac{\text{sensitivity}}{\left(1-\text{sensitivity}\right)}/\frac{\left(1-\text{specificity}\right)}{\text{specificity}}$$

The highest DOR value was obtained with 93.8% sensitivity and 88.2% specificity, where the diagnostic threshold was in the range of 6.5–7.5 mm. Therefore, we simply set the diagnostic threshold at 7 mm and prepared the DES sensor with the diagnostic electrodes accordingly (Figs 1B and 2B).

We validated the final sensor system, i.e., the system embedded with the glucose sensor and the DES sensor with the diagnostic electrodes. Normal and DES eyes of the rabbit were used, in which blood glucose levels were elevated using anesthesia (S2 Fig). To test the diagnostic property of the DES sensor, we measured the electrical resistance between the diagnostic electrodes. The measurable and infinite values of resistance indicated the normal and DES eyes, respectively. As shown in Table 1, the sensor system exhibited 91.7% sensitivity and 83.3% specificity in DES diagnosis for a total of 24 testing conditions. This also revealed a properly high accuracy of 87.5%, which was calculated by the following equation [33, 34]:

$$\text{Accuracy}\;(\text{\%})=\frac{\#\;\text{of}\;\text{true}\;\text{positive}+\#\text{of}\;\text{true}\;\text{negative}}{\#\;\text{of}\;\text{all}\;\text{testing}\;\text{conditions}}\times 100$$

**Table 1:** *In vivo* feasibility evaluation for DES diagnosis with the sensor system.

	DES animals	Normal animals
DES positive	11	2
DES negative	1	10

A total of 12 eyes were used for each of the animal groups, where each of the 4 eyes were measured at 0, 30, and 60 minutes after anesthesia.

**Fig 6:**
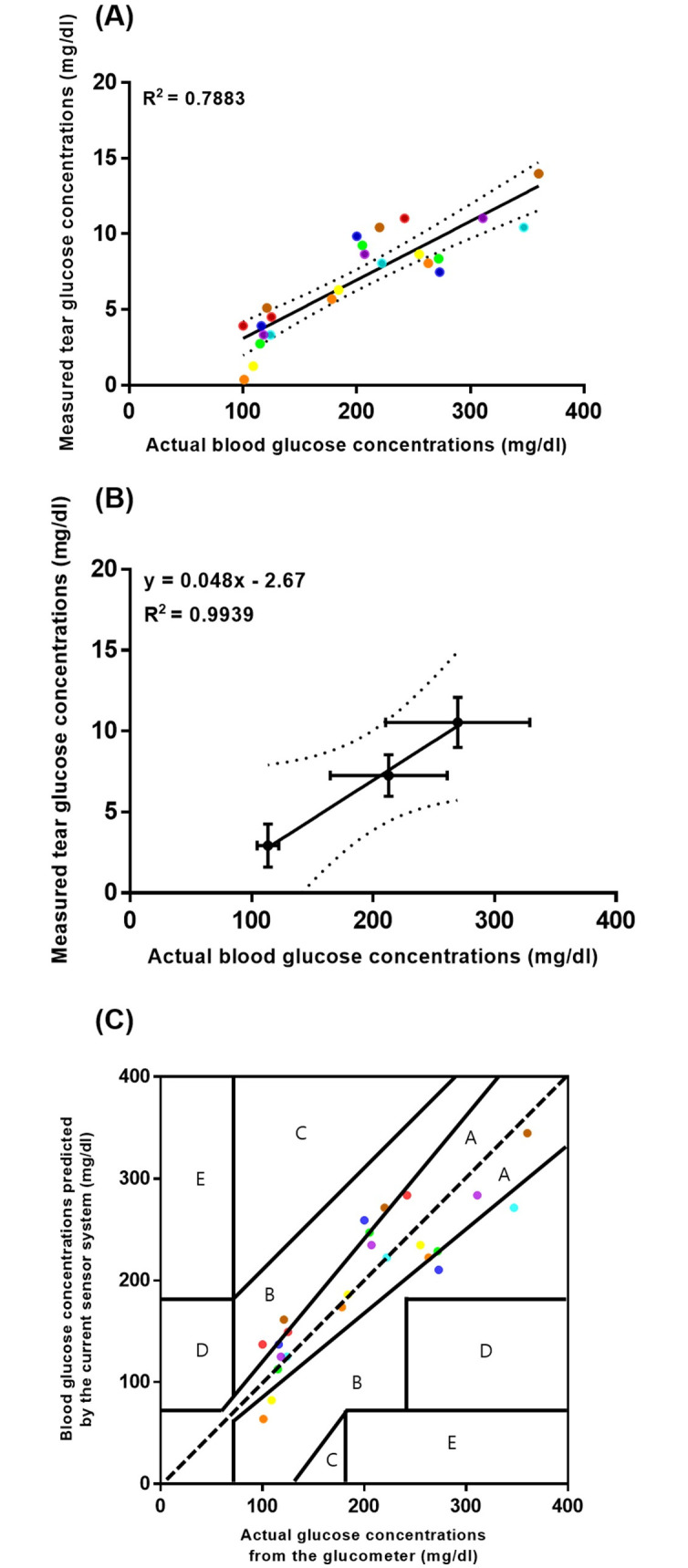
*In vivo* evaluation of the glucose sensor in the sensor system. (A) Plots between the tear glucose concentrations measured by the sensor system and obtained with the glucometer. Dotted lines indicate 95% confidence bands. The data points were color-coded to indicate each of the individual animals. (B) Plots between the average values of glucose concentration in tear and blood obtained at each time point of tear collection after anesthetic injection. Dotted lines indicate 95% confidence bands. (C) Clarke’s error grid analysis on the blood glucose concentrations predicted by the sensor system based on the levels actually measured using a clinically approved glucometer. The data points were color-coded to indicate each of the individual animals.

We also tried to estimate the glucose concentration in blood via the measurement of tears using our system. As shown in Fig 6A, there was a linear correlation between the glucose concentrations in tear and blood, but the linearity was not very high (R^2^ = 0.7883). These scattered data points were attributed to the inherent differences between individually tested animals, as reported in previous studies [19, 22, 23]. However, when the average values obtained at each sampling time were plotted (Fig 6B) [19, 22], there was a strong linear correlation (R^2^ = 0.9939), which also showed a statistically significant difference between the values at each sampling time (*P* < 0.05).

We then tested the reliability of our sensor system for the measurement of blood glucose concentrations. We first obtained the values of the predicted blood glucose concentrations, which were calculated from the tear glucose concentrations measured with the current sensor system, using the equation generated from Fig 6B. The predicted values were compared with the actual blood glucose concentrations obtained by the commercially available glucometer. When plotted in a Clarke error grid (Fig 6C), all data points were placed within zones A and B, with approximately 67% of data points in zone A, which suggests that the current sensor system was fairly acceptable to estimate the blood glucose concentrations compared with the clinically approved glucometer [19, 35, 36]. Multiple data points around at 100 mg/dl were ascribed to the normal glucose concentration often observed with the animals herein before anesthetic injection. With the tip design used in this work [21], the sensor system herein did not appear to damage the IPC of rabbit eyes after multiple preocular applications (S4 Fig).

## Discussion

Diabetes and DES are two closely related chronic diseases that require a continuous lifetime care together [3–6, 37]. For diabetes, blood glucose levels are recommended to be measured multiple times a day, where the blood is withdrawn somewhat invasively by pricking the fingertips. The volume of tear fluid available in the preocular space is often considered a determinant of DES. However, the collection of tear fluid is not easy, and it is often performed by the healthcare professionals [13, 32].

Therefore, we propose a noninvasive ease-of-use sensor system as a potential means for the self-monitoring of diabetes and DES. Instead of conventional needled blood withdrawal, the system herein collected basal tear fluid as a medium for the measurement of glucose concentrations [20, 22]. To diagnose DES, the system in this work also measured the volume of basal tear fluid as performed with the Schirmer’s test in clinical settings [38, 39]. Therefore, the contact tip of the sensor system was shaped to not irritate the eye surface during the collection of basal tear fluid (S4 Fig) [21, 23], and it was also purposed to contact the IPC, which is a less sensitive eye surface. Thus, when the sensor system was applied to the IPC, the upper eye lid could be closed to minimize any possible effect of tear break-up time. The minimized production of reflex tears during collection prevents the dilution of glucose in tear fluid, which led to the high accuracy in measurement (Fig 6). For the same reason, there could be an apparent threshold in the volume of collected tear fluid between the normal and DES eyes (Fig 5).

However, the amount of basal tear fluid available in the preocular space is very small, at approximately 7.0 ± 2.0 μl in humans [40], and the tear film thickness at the cornea was reported to be very thin [41]. Therefore, our sensor system was applied to the IPC of the eye, where more tear fluid would be present. Importantly, the sensor system was also designed to work with a small volume of about 0.75 μl tear fluid for glucose measurement and DES diagnosis (0.4 and about 0.35 μl for glucose and DES sensors, respectively). The sensor system was enabled with concurrent, parallel collection of tear fluid into two distinct sensors within a relatively short time (~ 10 s), which was expedited via capillary force through a microchannel made of a hydrophilic material. For this reason, the DES sensor was expected to be not very sensitive to the tear collection time difference of about 1 s (S5 Fig). However, to be more accurate, the user instruction regarding to the tear collection time may need to be properly provided. In many previous studies, to obtain tear fluids sufficient for measurements, the eye surface was stimulated to intentionally induce reflex tear generation [42–44], or tear fluid was collected for a longer duration, i.e., minutes [19], which would cause measurement inaccuracies and eye irritation.

For actual translational applications, our sensor system requires improvement. To estimate blood glucose concentrations more accurately, the inherent differences in correlations between the glucose concentrations in tear and blood between individuals need to be considered (Fig 6A) [19, 22, 23], which could also be ascribed to the variation in their temporal change [23, 45]. Therefore, the glucose sensor herein may need to be calibrated for each of the individual users to improve the accuracy. We set a diagnostic threshold based on the data obtained from animal models. Therefore, a new threshold must be set and confirmed for DES diagnosis based on clinical human studies.

A proper reading device also needs to be developed as an interface for the user’s convenience. Both sensors in the system were readable with electrical signals. Therefore, we could envision a single entity that combines two electrical signal readers. For example, the electrochemical glucose sensor herein could be read to give tear glucose concentrations in the same way as the conventional blood glucometer [30], which may just need calibration to estimate and display the glucose concentrations in blood. The reading of electrical resistance in the DES sensor, i.e., the measurable or infinite values, would distinguish and display the presence of DES conditions via the implementation of a simple electrical circuit [46].

In conclusion, we propose a sensor system that has a potential for monitoring diabetes and DES conditions via concurrent measurements of tear fluid. With a noninvasive shape of the preocular contact tip, the sensor system can collect and deliver basal tear fluid into the embedded glucose and DES sensors without damaging the eye tissues. Even with a small amount of collected tear fluid, the sensors with the proposed design estimated blood glucose levels and determined the DES conditions with acceptable accuracy. Therefore, we conclude that our preocular sensor system provides an alternative, minimally invasive strategy for the chronic management of diabetes and DES.

## Supporting information

S1 Fig *In vitro* setup for preparation of tear films. (A) Schematic description of the setup. (B) Thickness profiles of tear films prepared under two different flow rates, simulating the normal and DES conditions, respectively. The images were drawn by the authors using Solidworks (SOLIDWORKS Standard 2017 Research, Dassault Système, Vélizy-Villacoublay, France).
(TIF)

S2 Fig Profiles of *in vivo* animal models. (A) Schirmer’s test scores to induce dry eyes for the DES animal group. (B) Change in blood glucose concentration after subcutaneous injection of a cocktail of xylazine and ketamine.
(TIF)

S3 Fig Plots between the measured electrical current and known glucose concentrations in normal and DES films obtained under in vitro environments. (TIF)

S4 Fig Fluorescent images obtained from the IPC of fluorescein-stained rabbit eye. After three times of applications of the sensor system to the same eye, the tissue damage on IPC was examined, following the previous protocol.^1^ Briefly, a 5-μl drop of 0.25% w/v fluorescein sodium solution was instilled in the eye and after 5 min, the eye was washed thoroughly with normal saline to remove excess fluorescein solution. Then, a fluorescent image of the IPC surface was obtained, using a camera (Galaxy Note 9, Samsung, Seoul, Korea) equipped with the excitation (475 nm) and emission (542 nm) light filters (Thorlabs, Newton, NJ, USA). There was no visible staining on the IPC surface after multiple applications of our sensor system, suggesting no apparent tissue damage. In contrast, a stained region was clearly observed when the IPC was in contact with an intact Accu-Chek strip.
(TIF)

S5 Fig Infiltration length of fluids collected into the DES sensor under *in vitro* simulated environments at the difference of ±1 s based on 10 s collection time. The infiltration length was not statistically significantly different among the DES or normal film condition, respectively; however, the fluid collected for 11 s under the DES film condition was significantly smaller than that collected for 9 s under the normal film condition, suggesting that the difference of ±1 s collection time would be able to distinguish the normal and dry eye conditions. **** *P* < 0.0001.
(TIF)

S1 Material (DOCX)

S2 Material (DOCX)
