# Supplementary figures and images for: Preocular sensor system for concurrent monitoring of glucose levels and dry eye syndrome using tear fluids
Source: PLoS One. 2020 Oct 7;15(10):e0239317. doi: 10.1371/journal.pone.0239317 (PMC7540859; doi:10.1371/journal.pone.0239317)

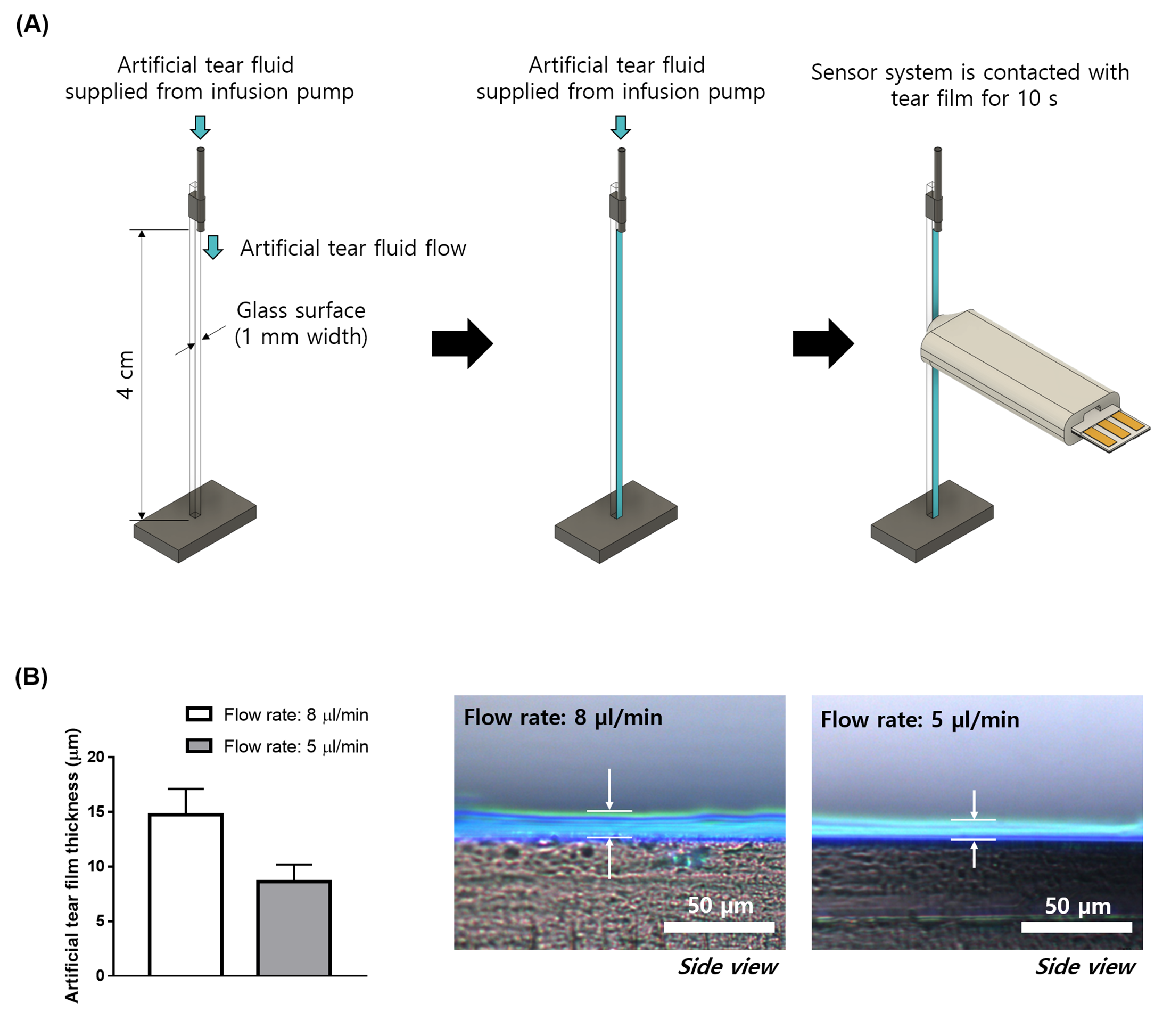

Supplement: S1 Fig — (A) Schematic description of the setup. (B) Thickness profiles of tear films prepared under two different flow rates, simulating the normal and DES conditions, respectively. The images were drawn by the authors using Solidworks (SOLIDWORKS Standard 2017 Research, Dassault Système, Vélizy-Villacoublay, France). (TIF) [file pone.0239317.s001.tif]

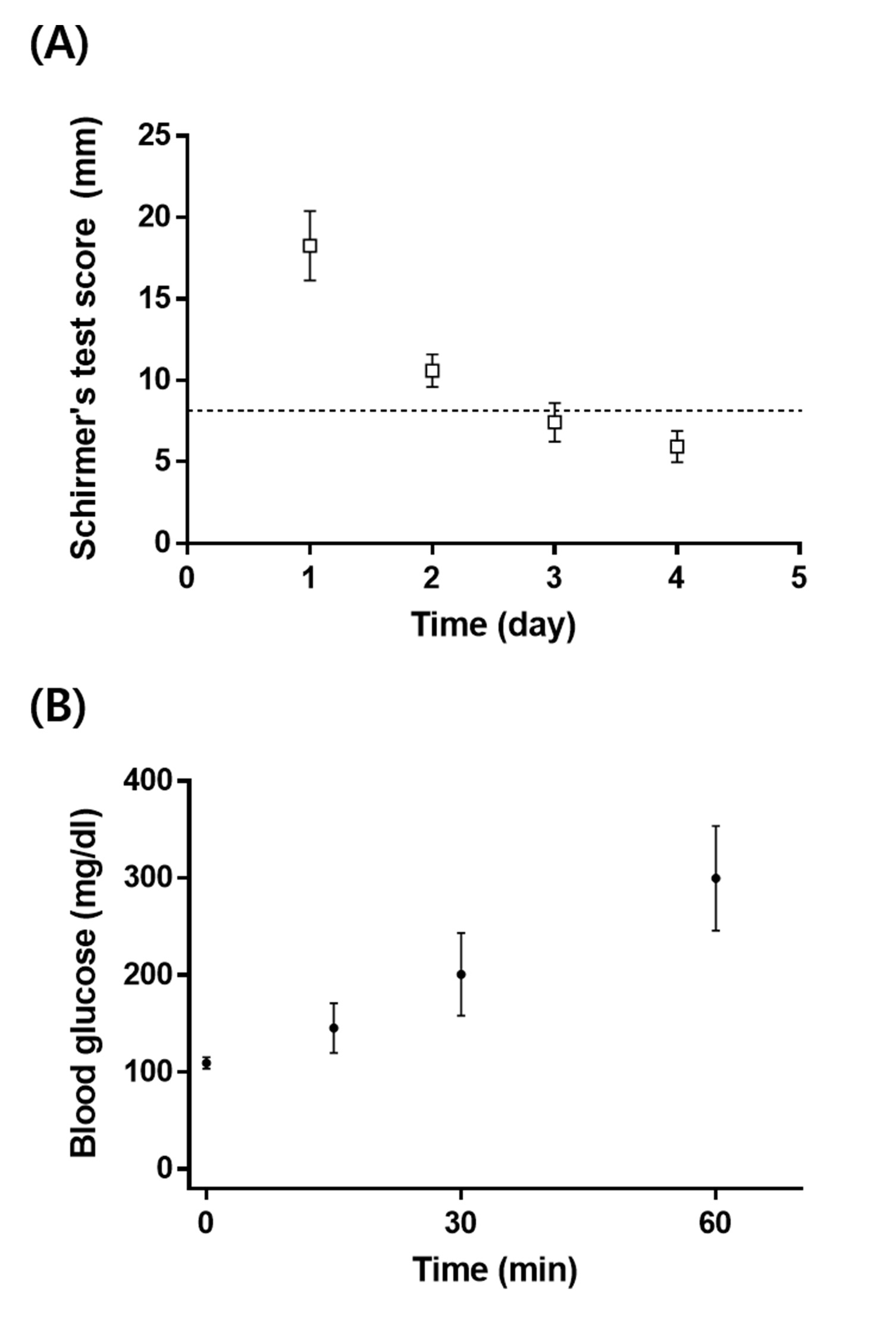

Supplement: S2 Fig — (A) Schirmer’s test scores to induce dry eyes for the DES animal group. (B) Change in blood glucose concentration after subcutaneous injection of a cocktail of xylazine and ketamine. (TIF) [file pone.0239317.s002.tif]

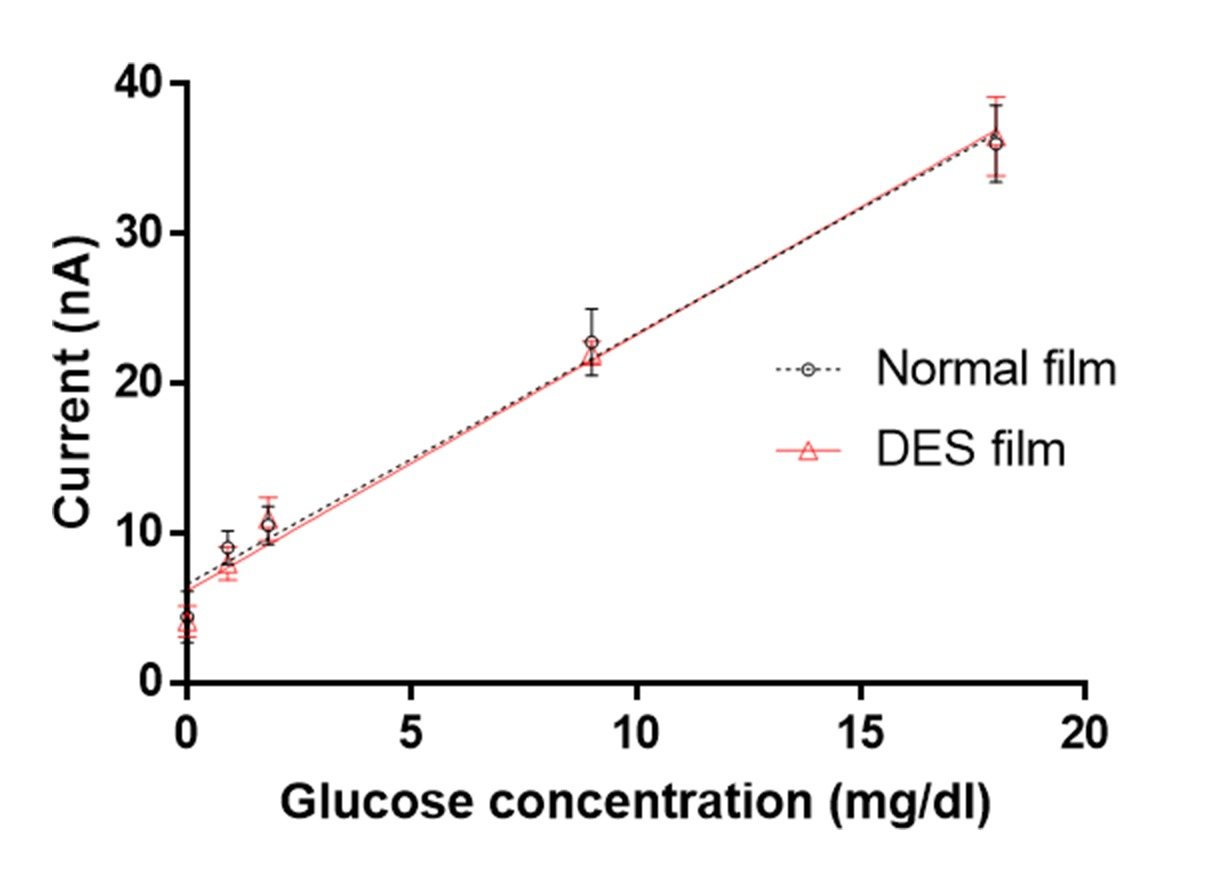

Supplement: S3 Fig — (TIF) [file pone.0239317.s003.tif]

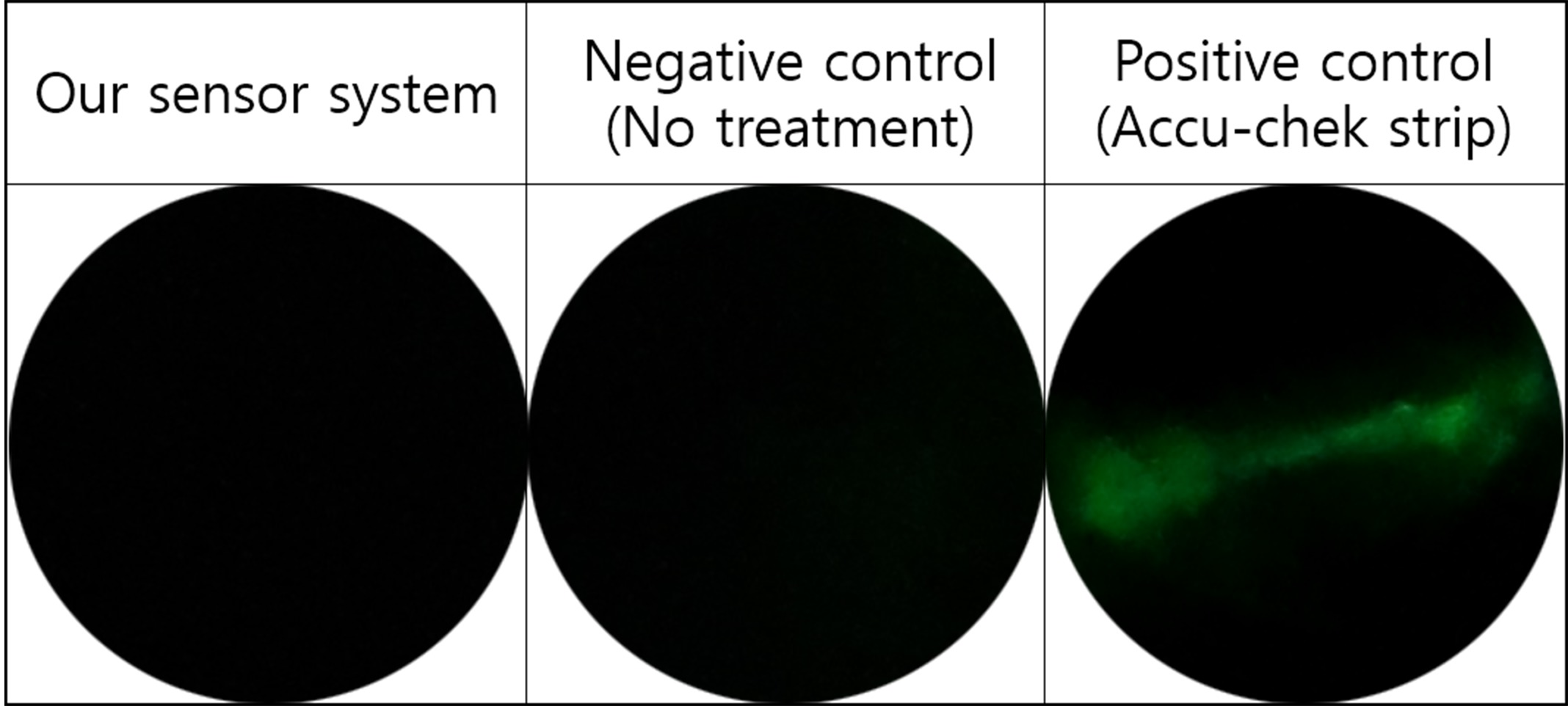

Supplement: S4 Fig — After three times of applications of the sensor system to the same eye, the tissue damage on IPC was examined, following the previous protocol.1 Briefly, a 5-μl drop of 0.25% w/v fluorescein sodium solution was instilled in the eye and after 5 min, the eye was washed thoroughly with normal saline to remove excess fluorescein solution. Then, a fluorescent image of the IPC surface was obtained, using a camera (Galaxy Note 9, Samsung, Seoul, Korea) equipped with the excitation (475 nm) and emission (542 nm) light filters (Thorlabs, Newton, NJ, USA). There was no visible staining on the IPC surface after multiple applications of our sensor system, suggesting no apparent tissue damage. In contrast, a stained region was clearly observed when the IPC was in contact with an intact Accu-Chek strip. (TIF) [file pone.0239317.s004.tif]

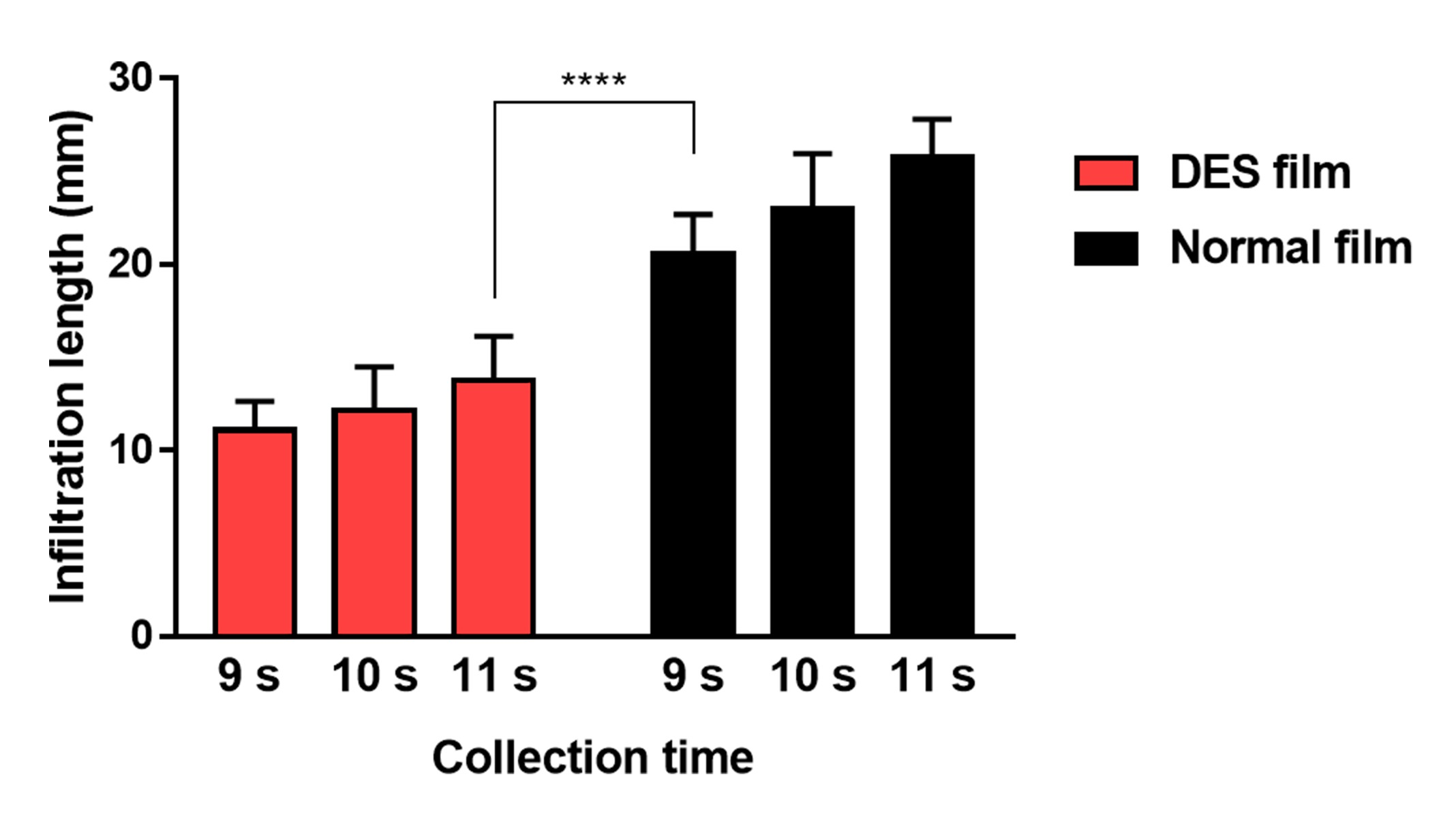

Supplement: S5 Fig — The infiltration length was not statistically significantly different among the DES or normal film condition, respectively; however, the fluid collected for 11 s under the DES film condition was significantly smaller than that collected for 9 s under the normal film condition, suggesting that the difference of ±1 s collection time would be able to distinguish the normal and dry eye conditions. **** P < 0.0001. (TIF) [file pone.0239317.s005.tif]
