# Supplementary material for: Preocular sensor system for concurrent monitoring of glucose levels and dry eye syndrome using tear fluids
Source: PLoS One. 2020 Oct 7;15(10):e0239317. doi: 10.1371/journal.pone.0239317 (PMC7540859; doi:10.1371/journal.pone.0239317)
Supplement: S1 Material — (DOCX) [file pone.0239317.s006.docx]

**Preocular sensor system for concurrent monitoring of glucose levels and dry eye syndrome using tear fluids**

Jae Hoon Han,^1¶^ Yong Chan Cho,^1¶^ Won-Gun Koh,^2^ and Young Bin Choy^1,3,4,*^

^1^ Interdisciplinary Program in Bioengineering, College of Engineering, Seoul National University, Seoul, Korea

^2^ Department of Chemical and Biomolecular Engineering, Yonsei University, Seoul, Korea

^3^ Institute of Medical & Biological Engineering, Medical Research Center, Seoul National University, Seoul, Korea

^4^ Department of Biomedical Engineering, Seoul National University College of Medicine, Seoul, Korea

* Corresponding author

E-mail: ybchoy@snu.ac.kr

^¶^These authors contributed equally as first authors to this work.

**Materials**

The constituent units of the proposed system were designed and drawn using SolidWorks (Dassault Système, Vélizy-Villacoublay, France) software. Materials for 3D printing (VeroClear) and supporting materials (SUP706) were purchased from Stratasys (Rehovot, Israel). Hydrophilic PET films (Melinex AF2) and adhesive tapes were obtained from 3M (Maplewood, MN, USA). D-(+)-Glucose, atropine sulfate, lysozyme, lactoferrin, fluorescein sodium and phosphate-buffered saline (PBS) were purchased from Sigma-Aldrich (St. Louis, MO, USA). The Accu-chek Performa test strips (Model Number: 04654011) and glucometer (Model Number: 04680456003) were purchased from Roche Diagnostics (Rotkreuz, Switzerland). Aluminum electrodes were purchased from Samjin (Seoul, Korea). A potentiostat (DY2113) and multimeter (D35) were obtained from Digi-Ivy (Austin, TX, USA) and OWON (Zhangzhou, China), respectively. Ketamine hydrochloride (Ketamine) and xylazine (Rompun) were purchased from Yuhan (Seoul, Korea) and Bayer (Leverkusen, Germany), respectively. Schirmer’s test strips were supplied from Katena (Parsippany, NJ, USA).

**Statistical Analysis**

The values of electrical current from the glucose sensor and the infiltration length from the DES sensor (W/O) are presented as the means ± standard deviations, and differences between groups were determined using Student’s t-test or one-way analysis of variance (ANOVA) followed by post-hoc Tukey's test for multiple comparisons using GraphPad Prism 7 (GraphPad Software, San Diego, CA, USA). Differences were considered statistically significant when *P* < 0.05.

**Supplementary figures**

**S1 Fig. *In vitro* setup for preparation of tear films.** (A) Schematic description of the setup. (B) Thickness profiles of tear films prepared under two different flow rates, simulating the normal and DES conditions, respectively. The images were drawn by the authors using Solidworks (SOLIDWORKS Standard 2017 Research, Dassault Système, Vélizy-Villacoublay, France).

**S2 Fig. Profiles of *in vivo* animal models.** (A) Schirmer’s test scores to induce dry eyes for the DES animal group. (B) Change in blood glucose concentration after subcutaneous injection of a cocktail of xylazine and ketamine.

**S3 Fig. Plots between the measured electrical current and known glucose concentrations in normal and DES films obtained under in vitro environments.**

**S4 Fig. Fluorescent images obtained from the IPC of fluorescein-stained rabbit eye.** After three times of applications of the sensor system to the same eye, the tissue damage on IPC was examined, following the previous protocol.^1^ Briefly, a 5-μl drop of 0.25% w/v fluorescein sodium solution was instilled in the eye and after 5 min, the eye was washed thoroughly with normal saline to remove excess fluorescein solution. Then, a fluorescent image of the IPC surface was obtained, using a camera (Galaxy Note 9, Samsung, Seoul, Korea) equipped with the excitation (475 nm) and emission (542 nm) light filters (Thorlabs, Newton, NJ, USA). There was no visible staining on the IPC surface after multiple applications of our sensor system, suggesting no apparent tissue damage. In contrast, a stained region was clearly observed when the IPC was in contact with an intact Accu-Chek strip.

**S5 Fig. Infiltration length of fluids collected into the DES sensor under *in vitro* simulated environments at the difference of ±1 s based on 10 s collection time.** The infiltration length was not statistically significantly different among the DES or normal film condition, respectively; however, the fluid collected for 11 s under the DES film condition was significantly smaller than that collected for 9 s under the normal film condition, suggesting that the difference of ±1 s collection time would be able to distinguish the normal and dry eye conditions. **** *P* < 0.0001.

**Supplementary Reference**

1. Lee SH, Cho YC, Choy YB. Noninvasive Self-diagnostic Device for Tear Collection and Glucose Measurement. Sci Rep. 2019;9(1):4747.
