# Supplementary material for: Preocular sensor system for concurrent monitoring of glucose levels and dry eye syndrome using tear fluids
Source: PLoS One. 2020 Oct 7;15(10):e0239317. doi: 10.1371/journal.pone.0239317 (PMC7540859; doi:10.1371/journal.pone.0239317)
Supplement: S2 Material — (DOCX) [file pone.0239317.s007.docx]

**Preocular sensor system for concurrent monitoring of glucose levels and dry eye syndrome using tear fluids**

Jae Hoon Han,^1¶^ Yong Chan Cho,^1¶^ Won-Gun Koh,^2^ and Young Bin Choy^1,3,4,*^

^1^ Interdisciplinary Program in Bioengineering, College of Engineering, Seoul National University, Seoul, Korea

^2^ Department of Chemical and Biomolecular Engineering, Yonsei University, Seoul, Korea

^3^ Institute of Medical & Biological Engineering, Medical Research Center, Seoul National University, Seoul, Korea

^4^ Department of Biomedical Engineering, Seoul National University College of Medicine, Seoul, Korea

* Corresponding author

E-mail: ybchoy@snu.ac.kr

^¶^These authors contributed equally as first authors to this work.

**Materials**

The constituent units of the proposed system were designed and drawn using SolidWorks (Dassault Système, Vélizy-Villacoublay, France) software. Materials for 3D printing (VeroClear) and supporting materials (SUP706) were purchased from Stratasys (Rehovot, Israel). Hydrophilic PET films (Melinex AF2) and adhesive tapes were obtained from 3M (Maplewood, MN, USA). D-(+)-Glucose, atropine sulfate, lysozyme, lactoferrin, fluorescein sodium and phosphate-buffered saline (PBS) were purchased from Sigma-Aldrich (St. Louis, MO, USA). The Accu-chek Performa test strips (Model Number: 04654011) and glucometer (Model Number: 04680456003) were purchased from Roche Diagnostics (Rotkreuz, Switzerland). Aluminum electrodes were purchased from Samjin (Seoul, Korea). A potentiostat (DY2113) and multimeter (D35) were obtained from Digi-Ivy (Austin, TX, USA) and OWON (Zhangzhou, China), respectively. Ketamine hydrochloride (Ketamine) and xylazine (Rompun) were purchased from Yuhan (Seoul, Korea) and Bayer (Leverkusen, Germany), respectively. Schirmer’s test strips were supplied from Katena (Parsippany, NJ, USA).

**Statistical Analysis**

The values of electrical current from the glucose sensor and the infiltration length from the DES sensor (W/O) are presented as the means ± standard deviations, and differences between groups were determined using Student’s t-test or one-way analysis of variance (ANOVA) followed by post-hoc Tukey's test for multiple comparisons using GraphPad Prism 7 (GraphPad Software, San Diego, CA, USA). Differences were considered statistically significant when *P* < 0.05.

**Supplementary Reference**

1. Lee SH, Cho YC, Choy YB. Noninvasive Self-diagnostic Device for Tear Collection and Glucose Measurement. Sci Rep. 2019;9(1):4747.
